# The Effects of Different Feedback Types on Learning With Mobile Quiz Apps

**DOI:** 10.3389/fpsyg.2021.665144

**Published:** 2021-05-31

**Authors:** Marco Rüth, Johannes Breuer, Daniel Zimmermann, Kai Kaspar

**Affiliations:** ^1^Department of Psychology, University of Cologne, Cologne, Germany; ^2^Data Archive for the Social Sciences, GESIS – Leibniz Institute for the Social Sciences, Cologne, Germany

**Keywords:** mobile learning, quiz apps, response feedback, learning performance, response certainty, self-assessment, semantic knowledge

## Abstract

Testing is an effective learning method, and it is the basis of mobile quiz apps. Quiz apps have the potential to facilitate remote and self-regulated learning. In this context, automatized feedback plays a crucial role. In two experimental studies, we examined the effects of two feedback types of quiz apps on performance, namely, the standard corrective feedback of quiz apps and a feedback that incorporates additional information related to the correct response option. We realized a controlled lab setting (*n* = 68, Study 1) and an unsupervised mobile setting (*n* = 150, Study 2). In the learning phase, participants used the quiz app and received feedback. They also completed a subsequent test as well as a follow-up test 1 week later by using the same quiz app. Irrespective of feedback type and setting, cognitive outcomes (quiz scores) and metacognitive outcomes (response certainty) increased similarly in the short term and long term. Feedback effects were not moderated by participants' overall response certainty during learning, their prior knowledge, and the difficulty of quiz items. Moreover, we found that participants perceived the quiz app to be similarly attractive, interesting, and enjoyable in both feedback conditions and that they spent slightly more time to process quiz items in the lab setting. We discuss these results in detail, including the role of moderating and mediating factors and prospects for further research and practice. Overall, our results underline that quiz apps are useful and effective tools that can support the acquisition and retention of semantic knowledge in different learning settings.

## Introduction

Mobile quiz applications (quiz apps) are popular and interactive software applications that have been used in television shows (Sperring and Strandvall, 2008), social media (Seebauer, 2014), and various educational settings. Educational settings include face-to-face (Hunsu et al., 2016), online (Boitshwarelo et al., 2017), and combined settings such as blended learning (Spanjers et al., 2015). In face-to-face settings, quizzes can function as audience response systems but with rather small effects on cognitive outcomes, as shown by meta-analytic results (Hunsu et al., 2016). Online/mobile settings include self-regulated learning processes (see Bjork et al., 2013) that can be supported by quiz apps. In general, quiz apps can function as formative learning tools that enable learners to (self-)monitor gains in achievement scores and that help them to develop cognitive processes related to self-regulated learning (McLaughlin and Yan, 2017). An effective and efficient way of learning with quiz apps is to process multiple-choice tests in single-choice format, which requires learners to identify the correct response option (target) from several options. This test format can support long-term retrieval (Roediger et al., 2011), but Roediger and Butler (2011, p. 20) also pointed out that “feedback enhances the benefits of testing”. Therefore, the quality of feedback implemented in quiz apps plays a crucial role when it comes to teaching and learning in online/mobile learning scenarios.

### In Search of Effective Feedback for Mobile Quiz Apps

Feedback is a core function of quiz apps that is fundamental for learning (Hattie and Timperley, 2007; Shute, 2008) and specifically relevant for self-regulated learning (Bjork et al., 2013), because feedback allows goal-oriented self-assessment with quiz apps (Nicol and Macfarlane-Dick, 2006). There are three common types of feedback implemented in multiple-choice quiz apps (for reviews, see Van der Kleij et al., 2011, 2015): First, knowledge of response feedback (KRF) only validates a response as correct or incorrect. Second, knowledge of correct response feedback (KCRF) labels the response as correct or incorrect and provides the correct answer, which can be considered the standard feedback type of multiple-choice quiz apps. Third, elaborated feedback (EF) subsumes several feedback types providing “additional information regarding the correctness of the response” (Van der Kleij et al., 2015, p. 6). Systematic reviews (Jaehnig and Miller, 2007; Van der Kleij et al., 2011) and a meta-analysis (Van der Kleij et al., 2015) have evaluated KRF as mostly ineffective, KCRF as moderately beneficial for obtaining lower-order learning outcomes, and EF as beneficial for obtaining both lower- and higher-order learning outcomes. However, since EF subsumes different feedback types providing additional information (see Shute, 2008; Van der Kleij et al., 2015), it is still unclear which subtype might be specifically effective for learning with quiz apps. A more recent meta-analysis on general effects of feedback on student learning indicated that “feedback is more effective the more information it contains” (Wisniewski et al., 2020, p. 12), but the large heterogeneity of feedback types and variance across interventions complicates drawing clear conclusions and inferring implementable solutions. Thus, for the purpose of our studies, we focused on using quiz apps to foster semantic general knowledge. Fostering such knowledge seems desirable, because it was found to predict students' performance in examinations (Furnham et al., 2009) and to be positively correlated with other facets of cognitive performance (Furnham et al., 2008; Schipolowski et al., 2014). Furthermore, we focused on immediate feedback, since it was found to be more effective than delayed feedback for lower-order learning outcomes such as knowledge acquisition (Shute, 2008; Van der Kleij et al., 2015) and since it was found to be processed for a longer duration than delayed feedback (Van der Kleij et al., 2012).

**Figure 1:**
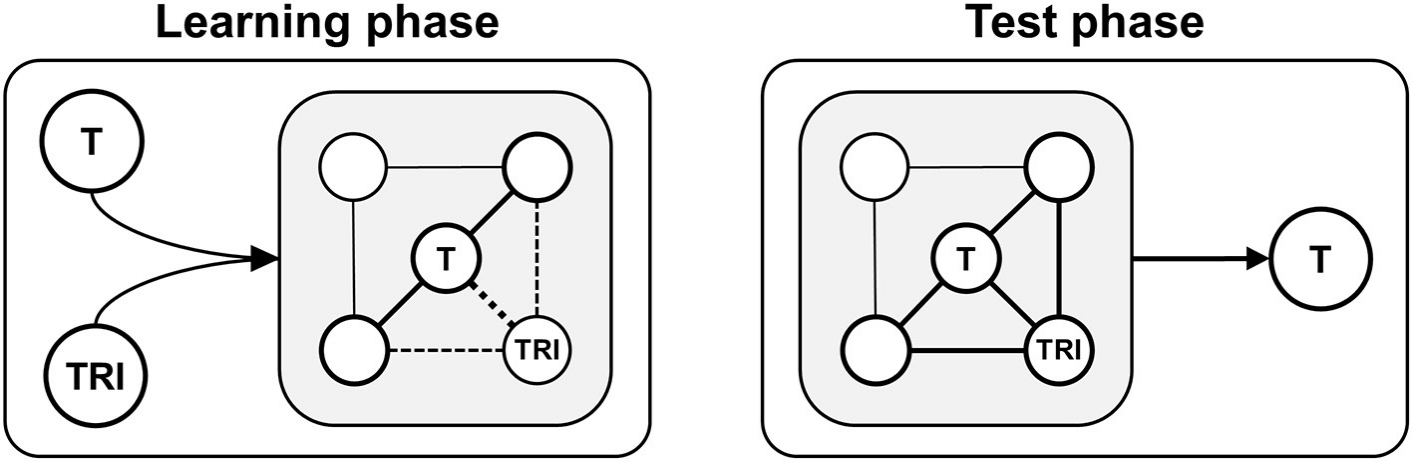
Schematic illustration of learning with information that is semantically associated with a correct response option (target-related information) and provided by attribute isolation feedback (AIF). Learning phase: A correct response (target, T) and target-related information (TRI) are processed, encoded, and integrated into (target-related) semantic memory networks. Memory traces are strengthened via activity within these networks (bold lines) and the formation of new semantic connections (dashed lines). Test phase: Processing of a target triggers activity in target-related semantic memory networks that store TRI (bold lines), which could increase the accuracy and certainty of recognizing and selecting the target.

While the standard KCRF only provides corrective feedback, several theoretical approaches suggest that providing additional target-related information (information semantically associated with a correct response option) could foster the acquisition and retention of knowledge, specifically the persistence of memories and the access to memories (cf. Roediger and Butler, 2011). Feedback that contains target-related information mostly corresponds to the so-called *attribute isolation feedback* (AIF), which is a subtype of EF that “presents information addressing central attributes of the target concept or skill being studied” (Shute, 2008, p. 160) and that “focuses learners on key components of the concept to improve general understanding of the phenomenon” (Mason and Bruning, 2001, p. 6). As illustrated by Figure 1, such feedback could increase the frequency (accuracy) of and confidence (certainty) in choosing targets: During learning phases (quiz sessions with feedback), target-related information could strengthen memory traces as it fosters in-depth processing of targets (and perhaps also of questions) (cf. Craik and Lockhart, 1972) and triggers activity in (target-related) semantic memory networks (Anderson, 1983). During test phases (quiz sessions without feedback), processing a target may trigger activity in target-related semantic memory networks (Anderson, 1983) so that access to target-related information and correct responding is facilitated. However, compared with the standard KCRF that is used in most quiz apps, AIF requires additional resources from learners (i.e., reading time and mental effort) as well as from teachers (i.e., time and effort needed for the creation and implementation of additional feedback information). Hence, besides the basic effectiveness of quiz apps, efficacy issues in terms of a cost–benefit ratio should also be considered when comparing feedback types. The present studies focused on a direct comparison between KCRF and AIF, including the potential impact of several moderator variables.

### The Present Studies

The present studies examined whether the standard feedback of mobile quiz apps (KCRF) and AIF yield different effects under controlled conditions in a lab setting (Study 1) and under less standardized but ecologically valid conditions in a more self-regulated mobile setting (Study 2). Based on previous results (Roediger et al., 2011; Van der Kleij et al., 2015), we expected to find short- and long-term cognitive benefits by using a quiz app that includes KCRF. In contrast to KCRF, AIF provides additional target-related information that can support encoding and retrieval of correct response options. Therefore, based on the abovementioned theoretical considerations (cf. Figure 1), we hypothesized that AIF would additionally foster associative memory processes, resulting in even higher knowledge gains:

H1: Participants show a higher increase in quiz performance (overall quiz score) in a subsequent test and a delayed follow-up test (1 week later) after having used a quiz app providing AIF compared with participants having used a quiz app providing KCRF.

Feedback can also support the correction of metacognitive errors, for instance, when learners give correct responses but have a low confidence in their responses (Butler et al., 2008). Response certainty provides a metacognitive estimate that reflects learners' retrieval confidence in self-regulated learning (see Bjork et al., 2013) and it can support learners to reflect on their answers (Nicol, 2007). Response certainty also indicates the extent to which learners understand the learning material and it is expected to be stored along with responses (for a review, see Mory, 2004). Indeed, both learning performance and response certainty were found to increase after retrieval tests (Barenberg and Dutke, 2019). In the present context of quiz apps, and in analogy to the hypothesized interaction effect in objective learning performance (H1), we expected:

H2: Participants show a higher increase in overall response certainty in a subsequent test and a delayed follow-up test after having used a quiz app providing AIF compared with participants having used a quiz app providing KCRF.

Particularly after low certainty responses, feedback should act as a critical instructional episode because “this is the theoretical point where large-scale elaborations should have their greatest positive effect” (Kulhavy and Stock, 1989, p. 297). Furthermore, learners receiving KCRF corrected fewer errors when initial answers were given with low certainty compared with initial answers given with high certainty (Butler et al., 2008; Griffiths and Higham, 2018). In contrast to KCRF, AIF provides additional information associated with the correct response option so that learners being less confident when completing a quiz could gain a more solid understanding of each question and the correct response option (cf. Kulhavy and Stock, 1989; Mory, 2004). Moreover, learners who complete a quiz with an overall high response certainty but with a low quiz score might also benefit more from AIF than from KCRF. In contrast, a simple validation of responses via KCRF might be sufficient for learners who perform well in terms of high quiz scores. Hence, we exploratively hypothesized:

H3: Participants' overall response certainty during learning moderates the effect of the received feedback type (KCRF vs. AIF) on participants' quiz performance in a subsequent test and a delayed follow-up test.

The effectiveness of feedback might also depend on learners' prior knowledge (Hattie and Timperley, 2007; Sitzman et al., 2014). Students with a low prior knowledge were found to benefit more from explanatory feedback than from corrective feedback (Moreno, 2004). Moreover, cognitive load can be induced by both elaborated feedback (cf. Swart et al., 2019) and task difficulty (Galy et al., 2012). Also, students with high cognitive load might need more guidance in learning (Moreno, 2007). Hence, we investigated if learners benefit differently from receiving KCRF and AIF given lower versus higher prior knowledge (H4) and given lower versus higher task difficulty (H5):

H4: Participants' prior knowledge, assessed in the learning phase, moderates the effect of the received feedback type (KCRF vs. AIF) on participants' quiz performance in a subsequent test and a delayed follow-up test.
H5: Task difficulty (i.e., the difficulty of quiz items) moderates the effect of the received feedback type (KCRF vs. AIF) on participants' quiz performance in a subsequent test and a delayed follow-up test.

Online/mobile quiz apps can be used anywhere and anytime. Learners have high flexibility in terms of self-regulated learning, given a free choice of location, time, and device. However, this could lead to distraction or multitasking (Zwarun and Hall, 2014) and thus longer item processing times compared to a controlled lab setting. Alternatively, participants in online/mobile settings might minimize their cognitive effort under uncontrolled conditions (Revilla and Ochoa, 2015) and this could result in shorter processing times compared to a lab setting. We therefore investigated whether the mean processing time in online/mobile settings is different than in a lab setting (H6a). We additionally examined whether feedback type (KCRF vs. AIF) moderates this difference in mean processing time (H6b):

H6a: The mean item processing time in the learning phase differs between a controlled lab setting (Study 1) and a mobile setting (Study 2).
H6b: The potential difference between Study 1 and Study 2 in terms of mean item processing time is moderated by feedback type (KCRF vs. AIF).

Finally, we considered users' subjective experiences when interacting with a quiz app. On the one hand, we examined whether users would find the quiz app utilizable and appealing. In this regard, instrumental (usability) as well as non-instrumental (aesthetics) qualities contribute to a positive user experience and were found to depend on structural and visual features of human–computer interfaces (Hamborg et al., 2014) and their overall quality (Kaspar et al., 2010). Hence, an effect of feedback type on user experience regarding quiz apps is conceivable. On the other hand, digital games are known for their positive effects on motivation and continuous use (Ryan et al., 2006) so that the game experience of quiz apps is of interest. However, previous research reported mixed results. Some authors reported effects of feedback type on motivation (e.g., Iterbeke et al., 2020) while others reported a general positive effect of quiz games on players' perceived enjoyment that was not moderated by feedback type (Tsai et al., 2015). Taken together, we examined whether a change in feedback type yields a different user experience (H7) and game experience (H8):

H7: Participants differently evaluate the user experience of a quiz app providing AIF compared with a quiz app providing KCRF.
H8: Participants differently evaluate the game experience of a quiz app providing AIF compared with a quiz app providing KCRF.

## Study 1

### Materials and Methods

#### Participants

We recruited 68 participants (*M*_age_ = 24.87, *SD*_age_ = 5.96; 41 female, 25 male, 1 other, 1 unspecified) via announcements in local university courses and randomly assigned them to the feedback conditions KCRF (*n* = 34) and AIF (*n* = 34). The minimum age required for participation was 18 years. We determined the sample size a priori, assuming a medium- to large-sized effect of *d* = 0.60 (cf. Cohen, 1988). This value was based on the averaged effect sizes of similar studies, which compared immediate KCRF with EF (*d* = 0.10, Merrill, 1987; *d* = 1.23, Kim and Phillips, 1991; *d* = 0.76, Pridemore and Klein, 1995; see Van der Kleij et al., 2015; and *d* = 0.10, Rüth et al., 2020). Such an effect size also seems desirable from a practical point of view because it requires more effort to create and implement AIF compared with KCRF. The required sample size was *n* = 62 for a 3 (quiz session) × 2 (feedback condition) repeated measures ANOVA, given a power of 0.80 and a significance level of 0.05 (GPower 3.1, Faul et al., 2007). All participants gave written informed consent and received a monetary compensation of 10€.

#### Design and Procedure

**Figure 2:**
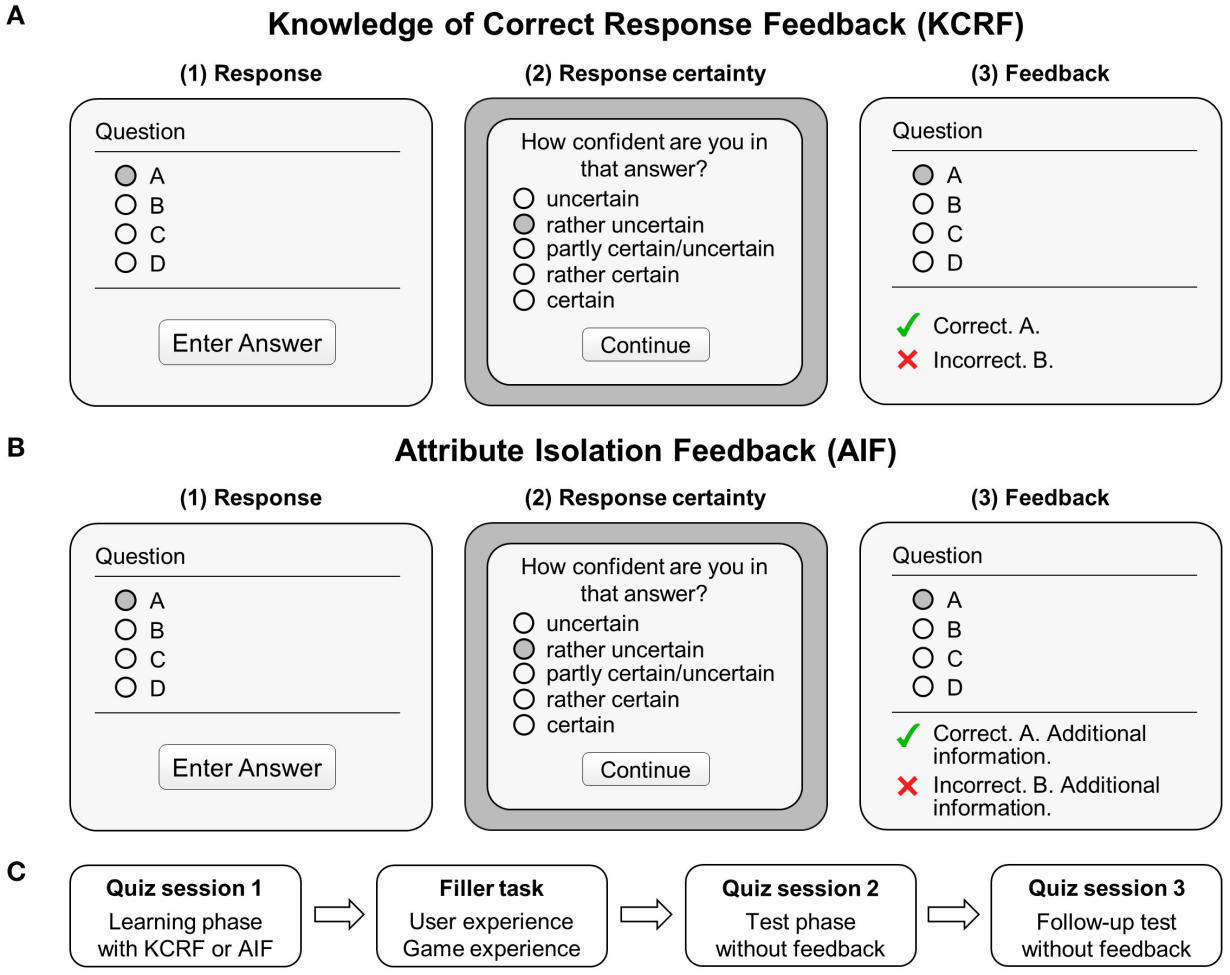
Learning with the quiz app. **(A)** Layout of the quiz app providing corrective feedback (KRFC). Participants selected a response, rated their response certainty, and received KCRF. The dark gray circles indicate exemplary responses and ratings. **(B)** The same quiz app was used to provide participants corrective feedback including additional information (attribute isolation feedback, AIF, see Table 1). **(C)** Design and procedure of Study 1 and Study 2.

We applied a 3 (quiz session) × 2 (feedback condition) experimental design. Quiz session (learning phase with feedback vs. test phase without feedback vs. follow-up test without feedback) was a within-subject factor and feedback condition (KCRF vs. AIF) was a between-subject factor. In quiz session 1 (learning phase), each participant completed a quiz with 40 quiz items in single-choice format (participants could select only one response option). Processing a quiz item included three steps: (1) selecting one out of four response options, (2) rating one's response certainty, and (3) receiving either KCRF or AIF (see Figure 2). Before the quiz started, these steps were demonstrated to all participants using sample material. Following the learning phase, participants rated their user experience and game experience, and provided sociodemographic information (age, gender, course of studies, and study semester). As shown in Figure 2, this phase simultaneously served as a filler task between the learning phase (quiz session 1) and the test phase (quiz session 2), resulting in a retention interval of approximately 5 min. Similar intervals were used in previous studies on feedback effects on semantic knowledge in online settings (Carpenter et al., 2008) and lab settings (Rüth et al., 2020). In the test phase, all participants responded to the same questions without receiving feedback. In an unannounced follow-up test (quiz session 3) that took place 1 week later, all participants again responded to the same questions without receiving feedback. While we initially communicated this appointment to the participants in the informed consent, we did not tell them that it would include a follow-up test. The order of quiz items was pseudo-randomized. There was no time limit, and the overall procedure was standardized. Participants completed all tasks using a laptop (Lenovo ThinkPad E540, 15.6-in. display) and an external computer mouse.

#### Quiz Materials

The quiz contained 40 single-choice quiz items on semantic (factual) knowledge. Initially, we selected eight questions for 12 common knowledge categories (history, literature, sports etc.), resulting in 96 questions with four response options each. Then, based on an online pre-study with an independent sample (*n* = 163), we assessed the difficulty of quiz items in terms of correct responses: We removed one quiz item hardly answered correctly at all (<10%) as well as eight quiz items answered correctly by almost all participants (>95%). Based on the percentage of participants who correctly answered a quiz item (item solution frequency), we then split the quiz items into three difficulty levels, resulting in 29 difficult (<33rd percentile), 29 moderate (between 33rd and 67th percentile), and 29 easy quiz items (>67th percentile). To avoid ceiling effects, we selected the most difficult (lowest solution frequency) quiz items from each difficulty level. The final quiz contained 13 easy (item solution frequency: *M* = 72.30%, *SD* = 3.82), 13 moderate (*M* = 48.37%, *SD* = 1.85), and 14 difficult quiz items (*M* = 22.48%, *SD* = 5.97) on eight knowledge categories. Item solution frequency differed significantly between the three difficulty levels, all *t*s ≥ 15.46, *p*s < 0.001, and *d*s ≥ 5.77. Similar to Irwing et al. (2001), correct and incorrect response options contained one or two words and were unambiguous. Finally, we formulated AIF for each quiz item, which consisted of a sentence that provided information related to the correct response option. For instance, regarding the question “What is the name of the author of ‘Robinson Crusoe’?”, “Daniel Defoe” is the correct response option (target). The corresponding target-related information (AIF) is: “The English author received worldwide fame through his novel ‘Robinson Crusoe’ and is regarded as a pioneer of English novels”. In this example, AIF provides several pieces of semantic information associated with the correct response option: “English author,” “worldwide fame,” and “pioneer of English novels”. These pieces of information may form new semantic nodes or relate to existing semantic clusters in learners' memory networks so that, for instance, “Daniel Defoe” is assigned to the category “English authors”. We provide more examples of quiz items and feedback information, including the logic for incorrect response options, in Table 1.

**Table 1:** Examples of quiz items for different knowledge categories and corresponding feedback messages provided after responding, and the logic for constructing incorrect response options.

**Quiz item and feedback messages**	**Logic for incorrect response options**
**Who went down in history as “Frederick the Great”? (history)**	Each incorrect response option also provided a name of a royal historical person
(a) Frederick II.	
(b) Frederick VII.	
(c) Frederick Barbarossa	
(d) Frederick IV.	
KCRF: Frederick II.	
AIF: Frederick II.—popularly known as Old Fritz—was king of Prussia from 1740 and king of Prussia and Elector of Brandenburg from 1772.	
**What is the name of the author of “Robinson Crusoe”? (literature)**	Each incorrect response option also provided a name of a novelist
(a) Daniel Defoe	
(b) Herman Melville	
(c) Mark Twain	
(d) Edgar Allen Poe	
KCRF: Daniel Defoe.	
AIF: Daniel Defoe. The English author received worldwide fame through his novel “Robinson Crusoe” and is regarded as a pioneer of English novels.	
**What is the name of one of the most famous cycling races in the world? (sports)**	Each incorrect response option provided a name similar to the correct response option
(a) Vuelta	
(b) Vuesta	
(c) Vulera	
(d) Volana	
KCRF: Vuelta.	
AIF: Vuelta. The Vuelta Ciclista a España—or Tour of Spain—is one of the three “Grand Tours” along with the Tour de France and the Giro d'Italia.	
**How many mountains on earth are higher than 8,000 m? (geography)**	Each incorrect response option provided a number around the correct number
(a) 14	
(b) 10	
(c) 18	
(d) 16	
KCRF: 14.	
AIF: 14. Ten of these so-called “eight-thousanders” are located in the Himalaya and four in the adjacent Karakoram.	
**Which name for a garment originates in the Indian language Hindi? (fashion)**	Each incorrect response option also provided a term for an item of clothing
(a) Pajama	
(b) Negligee	
(c) Parka	
(d) Caban	
KCRF: Pajama.	
AIF: Pajama. The word pajamas entered English in the nineteenth century, and since the beginning of the twentieth century in German has the meaning “sleeping suit”.	

*Each feedback message (KCRF: knowledge of correct response feedback; AIF: attribute isolation feedback) started with “Correct.” (following a correct response) or “Incorrect.” (following an incorrect response). Category names (e.g., history) were not displayed to participants*.

#### Measures

##### Quiz scores, response certainty, and item processing times

The quiz app (own development) recorded participants' quiz scores, response certainty, and item processing times (including reading the question and response options, selecting an option, evaluating the response certainty, and processing the response feedback). After selecting a response option, participants were asked to rate their response certainty (“How confident are you in that answer?”) based on a five-point rating scale (Mory, 2004) (“uncertain,” “rather uncertain,” “partly certain/uncertain,” “rather certain,” “certain”) (see Figure 2).

##### User experience and game experience

We assessed participants' user experience by means of the User Experience Questionnaire (UEQ, Laugwitz et al., 2008). The UEQ consists of 26 items measuring the app's attractiveness (Cronbach's α = 0.88), perspicuity (α = 0.60), dependability (α = 0.45), efficiency (α = 0.59), stimulation (α = 0.77), and novelty (α = 0.80). Each item is rated on a seven-point scale ranging from 1 to 7 where the scale's endpoints are labeled with semantically opposite attributes (e.g., “boring” vs. “exciting”).

To evaluate participants' game experience, we used three items measuring competence (α = 0.86, e.g., “I felt competent when playing the quiz game”), two items measuring game enjoyment (α = 0.77, e.g., “It is fun to play the quiz game”), and three items measuring game preference (α = 0.81, e.g., “I would like to play the game again in my leisure time”). These items were derived from the Player Experience of Need Satisfaction Questionnaire (Ryan et al., 2006) and from a study on use intention of a learning tool (Pedrotti and Nistor, 2014). The responses ranged from 1 (“do not agree”) to 5 (“strongly agree”) and were averaged to scales.

### Results

#### Feedback Effects on Quiz Performance Across Quiz Sessions (H1)

**Figure 3:**
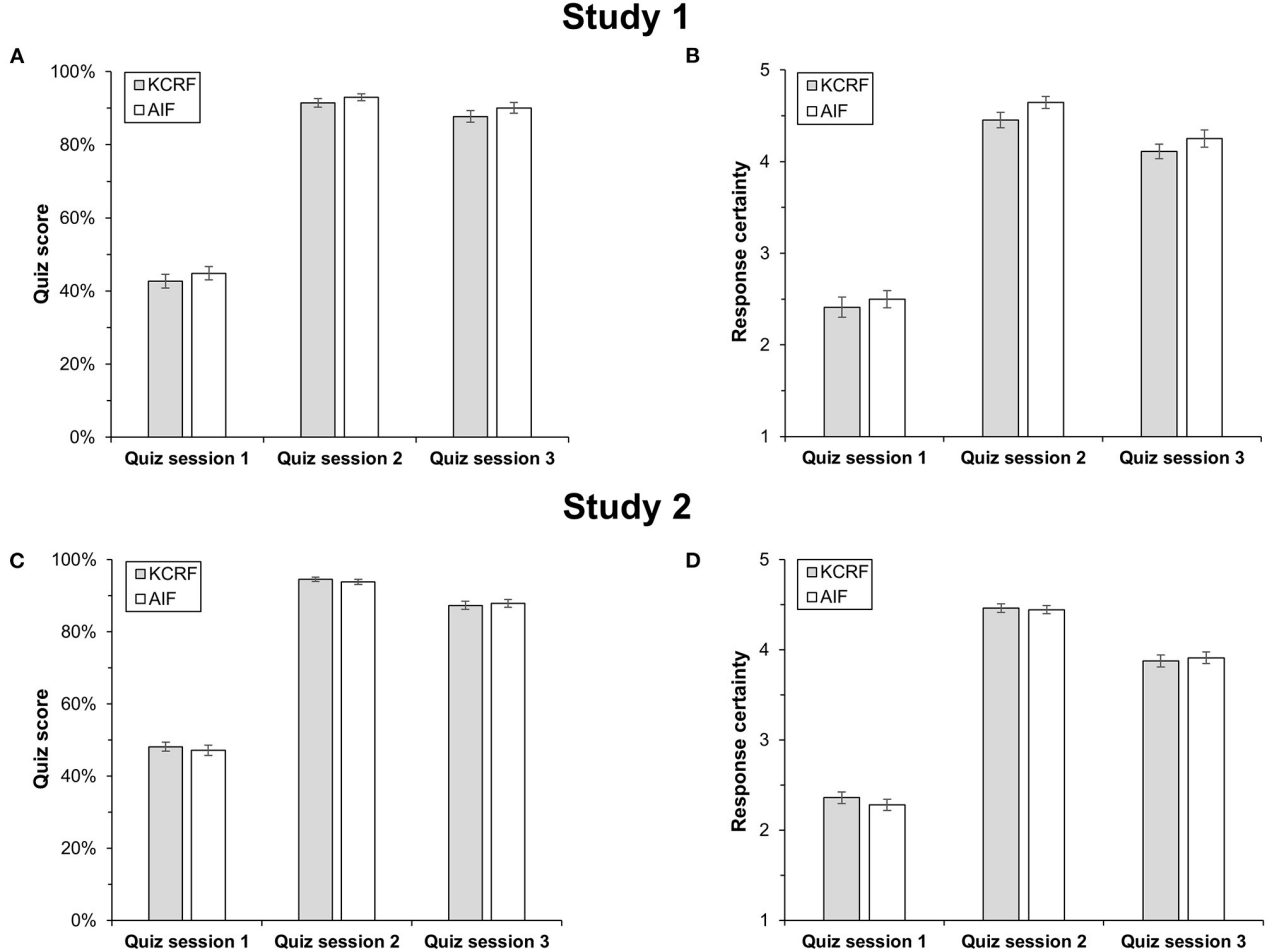
Quiz performance and response certainty following KCRF and AIF in Study 1 **(A,B)** and Study 2 **(C,D)**. Vertical lines indicate the standard error of the mean.

All analyses were performed with SPSS 27, and the Greenhouse–Geisser correction was applied for all ANOVAs. A 3 (quiz session) × 2 (feedback condition) ANOVA with quiz score as dependent variable showed a main effect of quiz session, *F*_(1.50,99.29)_ = 1149.36, *p* < 0.001, η_*p*_^2^ = 0.95, but no main effect of feedback condition, *F*_(1,66)_ = 1.41, *p* = 0.239, η_*p*_^2^ = 0.02. In contrast to H1, there was also no interaction between feedback type and quiz session, *F*_(1.50,99.29)_ = 0.07, *p* = 0.886, η_*p*_^2^ < 0.01. As shown in Figure 3A and supported by Bonferroni-adjusted *t*-tests, quiz scores increased from the learning phase (session 1) to the test phase (session 2), *p* < 0.001, *d* = 4.73, and decreased from the test phase to the follow-up test (session 3), *p* < 0.001, *d* = 0.55. Nonetheless, the performance gain was relatively long-lasting as quiz scores in session 3 were much higher than in session 1, *p* < 0.001, *d* = 4.16. Quiz scores in session 1 did not differ between KCRF and AIF, *t*(66) = −0.82, *p* = 0.417, *d* = 0.20, indicating that the random assignment of participants to feedback conditions was successful with regard to prior knowledge.

#### Feedback Effects on Response Certainty Across Quiz Sessions (H2)

A 3 (quiz session) × 2 (feedback condition) ANOVA with response certainty as dependent variable showed a main effect of quiz session, *F*_(1.64,108.19)_ = 772.73, *p* < 0.001, η_*p*_^2^ = 0.92 (Figure 3B), but no main effect of feedback condition, *F*_(1,66)_ = 1.68, *p* = 0.200, η_*p*_^2^ = 0.03. In contrast to H2, there was also no interaction, *F*_(1.64,108.19)_ = 0.43, *p* = 0.614, η_*p*_^2^ = 0.01. Response certainty was higher in sessions 2 and 3 compared with session 1, both *p*s < 0.001, *d*s ≥ 3.61, but it decreased from session 2 to session 3, *p* < 0.001, *d* = 1.04. Irrespective of the quiz session, response certainty and quiz scores were positively correlated in both feedback conditions, all *r*s ≥ 0.372, *p*s ≤ 0.030. In line with quiz scores, response certainty did not differ between KCRF and AIF in session 1, *t*(66) = −0.60, *p* = 0.550, *d* = 0.15.

#### Effect of Low vs. High Initial Response Certainty on Quiz Performance (H3)

Participants' mean response certainty reported in the learning session 1 served as a proxy for the overall certainty they initially perceived with respect to the quiz content. In order to test for a moderating role of this overall response certainty on the effectiveness of the feedback conditions on quiz performance, we focused on participants who reported a low (<33rd percentile, LRC group) or high mean response certainty (>67th percentile, HRC group) in session 1. The response certainty of the LRC and HRC groups differed significantly in both feedback conditions in session 1, *t*s ≥ 8.49, *p*s < 0.001, *d*s ≥ 3.44. A 3 (quiz session) × 2 (feedback condition) × 2 (response certainty group) ANOVA with quiz score as dependent variable showed a main effect of quiz session, *F*_(1.56,65.57)_ = 759.53, *p* < 0.001, η_*p*_^2^ = 0.95, and a main effect of response certainty group with higher test scores in HRC than LRC, *F*_(1,42)_ = 6.62, *p* = 0.014, η_*p*_^2^ = 0.14, but no main effect of feedback condition, *F*_(1,42)_ = 0.12, *p* = 0.727, η_*p*_^2^ < 0.01. Moreover, we found a significant interaction between quiz session and response certainty group, *F*_(1.56,65.57)_ = 4.57, *p* = 0.021, η_*p*_^2^ = 0.10: While quiz scores were higher in the HRC group than in the LRC group in session 1, *t*(44) = 3.32, *p* = 0.002, *d* = 0.98, there was no difference in session 2, *t*(36.05) = 1.73, *p* = 0.092, *d* = 0.52, and session 3, *t*(44) = 0.97, *p* = 0.340, *d* = 0.29. In both response certainty groups, quiz scores increased from session 1 to session 2 (*p*s < 0.001). Quiz scores decreased significantly from session 2 to session 3 in the high response certainty group, *t*(23) = −2.84, *p* = 0.009, *d* = 0.58, but not in the low response certainty group, *t*(21) = −1.95, *p* = 0.064, *d* = 0.42. Contrary to H3, all other interactions were non-significant, *F*s ≤ 0.79, *p*s ≥ 0.428, η_*p*_^2^ ≤ 0.02. Hence, participants' overall response certainty in the initial learning session did not moderate the effect of feedback type.

#### Feedback Effects on Quiz Performance Given Low vs. High Prior Knowledge (H4)

**Figure 4:**
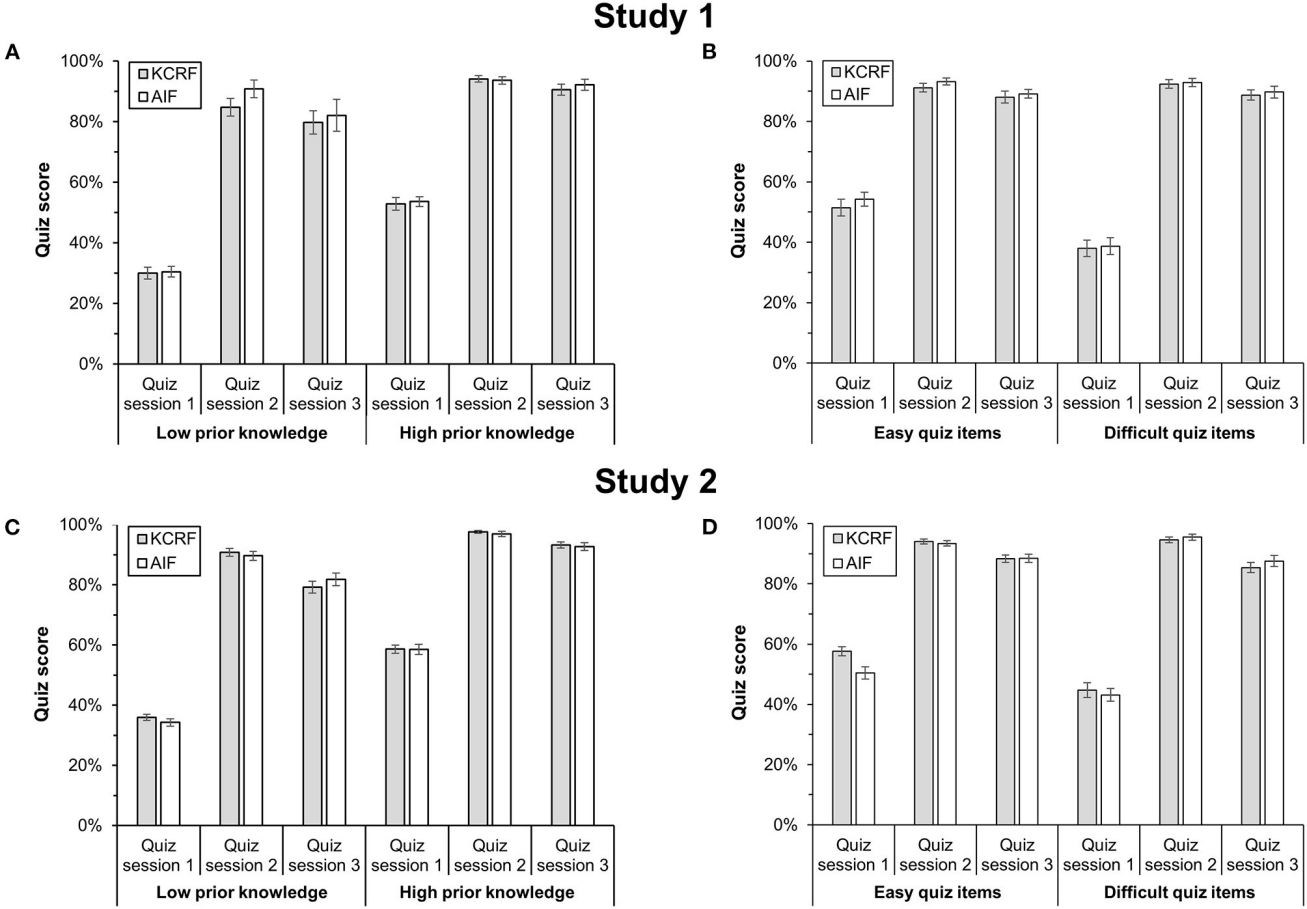
Moderating effects of participants' prior knowledge and the difficulty of quiz items on quiz performance when receiving KCRF and AIF in Study 1 and Study 2. **(A)** Quiz scores of participants with low prior knowledge and high prior knowledge in Study 1. **(B)** Quiz scores for easy and difficult quiz items in Study 1. **(C)** Quiz scores of participants with low prior knowledge and high prior knowledge in Study 2. **(D)** Quiz scores for easy and difficult quiz items in Study 2. Vertical lines indicate the standard error of the mean.

We divided participants into groups of low (<33rd percentile, LPK group) and high prior knowledge (>67th percentile, HPK group), based on quiz scores in session 1. Consequently, quiz scores of the LPK and HPK groups differed significantly in both feedback conditions in session 1, *t*s ≥ 7.48, *p*s < 0.001, *d*s ≥ 3.20. We performed a 2 (quiz session: 2 vs. 3) × 2 (feedback condition) × 2 (prior knowledge group) ANOVA with quiz score as dependent variable. As indicated in Figure 4A, we found a main effect of quiz session, *F*_(1,41)_ = 19.62, *p* < 0.001, η_*p*_^2^ = 0.32, and a main effect of prior knowledge, *F*_(1,41)_ = 14.17, *p* = 0.001, η_*p*_^2^ = 0.26, but no main effect of feedback condition, *F*_(1,41)_ = 1.20, *p* = 0.280, η_*p*_^2^ = 0.03. We also found an interaction between quiz session and prior knowledge, *F*_(1,41)_ = 4.30, *p* = 0.044, η_*p*_^2^ = 0.10: The HPK group outperformed the LPK group in sessions 2 and 3, with a smaller difference in session 2, *t*(17.83) = 2.85, *p* = 0.011, *d* = 1.10, than in session 3, *t*(19.08) = 3.27, *p* = 0.004, *d* = 1.22, irrespective of the feedback type received in session 1 (see Figure 4A). Notably, quiz scores decreased more from session 2 to session 3 in the LPK group, *t*(14) = −2.86, *p* = 0.013, *d* = 0.74, than in the HPK group, *t*(29) = −2.55, *p* = 0.016, *d* = 0.47. With respect to interaction hypothesis H4, we found no further interaction, *F*s ≤ 1.96, *p*s ≥ 0.169, η_*p*_^2^ ≤ 0.05.

#### Feedback Effects on Quiz Performance Depending on Difficulty of Quiz Items (H5)

In the pre-study, we categorized all quiz items as easy, moderate, or difficult, based on their respective solution frequency. However, there was only a small overlap in solution frequency between the pre-study and session 1 (30%), indicating sample differences. For the present analysis, we hence re-categorized all quiz items based on their solution frequency in session 1 as easy (>67th percentile) and difficult (<33rd percentile); observed quiz scores consequently differed between easy and difficult quiz items, *t*s ≥ 4.08, *p*s < 0.001, *d*s ≥ 0.70. Then, we performed a 2 (quiz session: 2 vs. 3) × 2 (feedback condition) × 2 (difficulty of quiz items) ANOVA with quiz score as dependent variable. We found a main effect of quiz session, *F*_(1,66)_ = 20.30, *p* < 0.001, η_*p*_^2^ = 0.24, but no main effect of the difficulty of quiz items, *F*_(1,66)_ = 0.44, *p* = 0.508, η_*p*_^2^ = 0.01, and no main effect of feedback condition, *F*_(1,66)_ = 0.42, *p* = 0.519, η_*p*_^2^ = 0.01. All interactions were non-significant, *F*s ≤ 0.26, *p*s ≥ 0.609, η_*p*_^2^ ≤ 0.01 (see Figure 4B). In other words, and with respect to H5, we found no moderating effect of the difficulty of quiz items.

#### User Experience and Game Experience (H7, H8)

As shown in Table 2, we found no difference in user experience between KCRF and AIF (H7). Participants' user experience was above the scale's midpoint for all rating dimensions in both feedback conditions, except for novelty. The game experience also did not differ between feedback conditions (H8). Participants' game enjoyment and game preference were above the scale's midpoint in both feedback conditions, whereas their perceived competence was rated below the scale's midpoint.

**Table 2:** Reported user experience and game experience in Study 1 and Study 2.

	**Study 1 (lab setting)**					**Study 2 (mobile setting)**				
	**KCRF^a^**	**AIF^a^**				**KCRF^b^**	**AIF^c^**			
**Measure**	**M (SD)**	**M (SD)**	***t***	***p***	***d***	**M (SD)**	**M (SD)**	***t***	***p***	***d***
**User experience**										
Attractiveness	4.65^**^ (1.12)	5.04^***^ (1.03)	−1.51	0.136	0.37	5.00^***^ (1.04)	4.75^***^ (1.15)	1.35	0.178	0.22
Perspicuity^d^	5.56^***^ (0.87)	5.69^***^ (0.98)	−0.59	0.559	0.14	5.36^***^ (0.89)	5.29^***^ (0.97)	0.44	0.663	0.07
Dependability^d^	4.40^**^ (0.73)	4.63^***^ (0.75)	−1.27	0.207	0.31	4.18^*^ (0.78)	4.04 (0.89)	1.02	0.310	0.17
Efficiency^d^	4.99^***^ (0.77)	5.22^***^ (0.86)	−1.19	0.237	0.29	4.99^***^ (0.74)	4.84^***^ (0.67)	1.30	0.196	0.21
Stimulation	4.77^***^ (1.05)	5.02^***^ (1.03)	−0.99	0.325	0.24	5.03^***^ (1.08)	4.80^***^ (1.27)	1.21	0.230	0.20
Novelty	3.67 (1.36)	3.88 (1.05)	−0.72	0.473	0.18	3.92 (1.14)	4.00 (1.15)	−0.41	0.680	0.07
**Game experience**
Competence	2.48^**^ (0.92)	2.90 (0.93)	−1.87	0.065	0.45	2.67^**^ (0.88)	2.42^***^ (0.91)	1.66	0.098	0.27
Game enjoyment	3.57^**^ (1.13)	3.82^***^ (0.94)	−0.99	0.324	0.24	3.78^***^ (1.01)	3.66^***^ (1.09)	0.66	0.512	0.11
Game preference	3.42^*^ (0.99)	3.59^**^ (0.94)	−0.71	0.478	0.17	3.46^***^ (1.04)	3.29^*^ (1.08)	0.95	0.346	0.16

*Values depict means (standard deviations). Asterisks indicate the results of one-sample t- tests comparing the mean with the scale's midpoint of 4 (user experience) or 3 (game experience). KCRF, knowledge of correct response feedback; AIF, attribute isolation feedback*.

a *n = 34;*

b *n = 83;*

c *n = 67;*

d *Scales' internal consistencies were below 0.60, that is, below the common threshold for acceptable internal consistencies of 0.70 (Tavakol and Dennick, 2011)*.

* *p < 0.05.*

** *p < 0.01.*

*** *p < 0.001*.

### Discussion

Contrary to our expectations, AIF did not lead to larger effects than KCRF on knowledge retention in terms of quiz scores (H1) and response certainty (H2). Quiz scores and response certainty were positively correlated and they substantially increased from the learning phase (session 1) to the test phase (session 2), with a slight decrease from the test phase to the follow-up test (session 3). Both feedback types were similarly effective, independent of participants' initial response certainty (H3) and prior knowledge (H4), and independent of whether easy or difficult quiz items were processed (H5). Hence, we did not find any support for corresponding moderation effects. Nonetheless, Study 1 revealed some interesting interactions: A significant interaction was found between quiz session and response certainty group as quiz performance was higher in the group of participants who reported high (compared with low) response certainty in session 1 (learning phase), but this effect was not observed in sessions 2 and 3. Moreover, participants with high prior knowledge, determined in the learning phase, showed a more temporally stable performance gain than participants with low prior knowledge, because their decrease in quiz performance from session 2 to session 3 was smaller. Participants' user experience (H7) and game experience (H8) were similar for both feedback types, with ratings above the scales' midpoints on most dimensions, indicating high instrumental and non-instrumental qualities of the app as well as high game enjoyment and game preference. Overall, using a quiz app with feedback in a learning session substantially increased performance and response certainty not only at short-term (session 2) but also long-term periods (session 3), irrespective of the feedback type.

## Study 2

Learners using mobile quiz apps can benefit from advantages of mobile learning, for instance, by realizing their preferred way of learning. This includes choosing a preferred space, time, and device (cf. Grant, 2019). Accordingly, the purpose of Study 2 was to validate our findings from a controlled lab setting (Study 1) in a more realistic and unsupervised online setting. In Study 2, participants were free to choose their preferred location, time, and device when using the quiz app.

### Materials and Methods

#### Participants

Participants were recruited online via mailing lists and social networks. A total of 189 participants (with a minimum age of 18 years) gave written informed consent and completed all three quiz sessions. We excluded 39 participants due to implausible processing times in at least one of the three quiz sessions: Based on the mean item processing time, we identified participants being very fast so that they could hardly have read the questions and response options carefully (<5th percentile), and participants being very slow so that they were presumably distracted or stopped the quiz intermediately (>95th percentile) (Kaspar et al., 2015). Eventually, we analyzed the data of 150 participants (*M*_age_ = 26.10, *SD*_age_ = 6.85; 125 female, 24 male, 1 other) who provided a complete and valid data set and were randomly assigned to the KCRF (*n* = 83) and AIF condition (*n* = 67) by the quiz app. A monetary compensation of 10€ was given to 40 participants by means of a lottery.

#### Design and Procedure

The design and procedure of Study 2 were identical to Study 1, except that participants in Study 2 were free to choose a location, time, and device to complete the study. Most participants completed the learning and test phase at home (83.33%), but also at university (10.67%), at work (4.00%), or on the way (2.00%). Similarly, the follow-up test was mostly completed at home (79.33%), at university (10.67%), at work (5.33%), or on the way (4.00%), while one participant gave no response (0.67%). We used the same quiz items and measures as in Study 1, with slightly different internal consistencies of the UEQ's scales (attractiveness: α = 0.92; perspicuity: α = 0.50; dependability: α = 0.52; efficiency: α = 0.45; stimulation: α = 0.86; novelty: α = 0.79) and of the scales that we used to measure game experience (competence: α = 0.90; game enjoyment: α = 0.79; game preference: α = 0.88). Scales were again formed by averaging the corresponding items.

### Results

#### Feedback Effects on Quiz Performance Across Quiz Sessions (H1)

Replicating the results of Study 1, a 3 (quiz session) × 2 (feedback condition) ANOVA with quiz score as dependent variable showed a main effect of quiz session, *F*_(1.62,239.41)_ = 2450.23, *p* < 0.001, η_*p*_^2^ = 0.94, no main effect of feedback condition, *F*_(1,148)_ = 0.09, *p* = 0.770, η_*p*_^2^ < 0.01, and no interaction, *F*_(1.62,239.41)_ = 0.66, *p* = 0.489, η_*p*_^2^ < 0.01 (Figure 3C). Again, quiz scores were higher in session 2, *p* < 0.001, *d* = 4.68, and session 3, *p* < 0.001, *d* = 4.12, than in session 1, but decreased from session 2 to session 3, *p* < 0.001, *d* = 1.04. Quiz scores in session 1 did not differ between KCRF and AIF, *t*(148) = 0.53, *p* = 0.594, *d* = 0.09.

#### Feedback Effects on Response Certainty Across Quiz Sessions (H2)

Replicating the results of Study 1, a 3 (quiz session) × 2 (feedback condition) ANOVA with response certainty as dependent variable showed a main effect of quiz session, *F*_(1.74,257.14)_ = 1777.00, *p* < 0.001, η_*p*_^2^ = 0.92, but no main effect of feedback condition, *F*_(1,148)_ = 0.08, *p* = 0.777, η_*p*_^2^ < 0.01, and no interaction, *F*_(1.74,257.14)_ = 1.17, *p* = 0.308, η_*p*_^2^ = 0.01 (Figure 3D). Response certainty increased from session 1 to sessions 2 and 3, *p*s < 0.001, *d*s ≥ 4.38, and decreased from session 2 to session 3, *p* < 0.001, *d* = 1.09. Again, response certainty and quiz scores were positively correlated in both feedback conditions irrespective of quiz session, *r*s ≥ 0.410*, p*s ≤ 0.001. The response certainty observed in session 1 did not differ between KCRF and AIF, *t*(148) = 0.87, *p* = 0.385, *d* = 0.14.

#### The Effect of Low vs. High Initial Response Certainty on Quiz Performance (H3)

As in Study 1, we focused on participants with low (<33rd percentile, LRC group) and high mean response certainty (>67th percentile, HRC group) in session 1. In both feedback conditions, response certainty was higher in the HRC group than in the LRC group, *t*s ≥ 12.07, *p*s < 0.001, *d*s ≥ 3.47. A 3 (quiz session) × 2 (feedback condition) × 2 (response certainty group) ANOVA showed a main effect of quiz session on quiz scores, *F*_(1.63,159.68)_ = 1726.61, *p* < 0.001, η_*p*_^2^ = 0.95, a main effect of response certainty group, *F*_(1,98)_ = 22.92, *p* < 0.001, η_*p*_^2^ = 0.19, but no main effect of feedback condition, *F*_(1,98)_ < 0.01, *p* = 0.959, η_*p*_^2^ < 0.01. We again found a significant interaction between quiz session and response certainty group, *F*_(1.63,159.68)_ = 14.34, *p* < 0.001, η_*p*_^2^ = 0.13: While quiz scores were higher in the HRC group than in the LRC group in session 1, *t*(100) = 5.58, *p* < 0.001, *d* = 1.11, this difference was smaller in session 2, *t*(78.64) = 2.85, *p* = 0.006, *d* = 0.58, and session 3, *t*(78.97) = 3.26, *p* = 0.002, *d* = 0.66. In both response certainty groups, quiz scores increased from session 1 to session 2 and decreased from session 2 to session 3 (*p*s < 0.001). As in Study 1 and contrary to H3, all other interactions were non-significant, *F*s ≤ 1.72, *p*s ≥ 0.193, η_*p*_^2^ ≤ 0.02.

#### Feedback Effects on Quiz Performance Given Low vs. High Prior Knowledge (H4)

As in Study 1, quiz scores of participants with low (<33rd percentile, LPK group) and high prior knowledge (>67th percentile, HPK group) differed in both feedback conditions, *t*s ≥ 11.94, *p*s < 0.001, *d*s ≥ 3.38. A 2 (quiz session: 2 vs. 3) × 2 (feedback condition) × 2 (prior knowledge group) ANOVA with quiz score as dependent variable revealed a main effect of quiz session, *F*_(1,103)_ = 141.48, *p* < 0.001, η_*p*_^2^ = 0.58, a main effect of prior knowledge group, *F*_(1,103)_ = 68.66, *p* < 0.001, η_*p*_^2^ = 0.40, but no main effect of feedback condition, *F*_(1,103)_ < 0.01, *p* = 0.975, η_*p*_^2^ < 0.01. We again found an interaction between quiz session and prior knowledge group, *F*_(1,103)_ = 20.84, *p* < 0.001, η_*p*_^2^ = 0.17: As shown in Figure 4C, the HPK group outperformed the LPK group in sessions 2 and 3, with a smaller difference in session 2, *t*(66.88) = 6.58, *p* < 0.001, *d* = 1.36, than in session 3, *t*(71.14) = 7.82, *p* < 0.001, *d* = 1.60. Again, quiz scores decreased more from session 2 to session 3 in the LPK group, *t*(47) = −9.52, *p* < 0.001, *d* = 1.37, than in the HPK group, *t*(58) = −6.73, *p* < 0.001, *d* = 0.88. As in Study 1, and with respect to interaction hypothesis H4, all other interactions were again non-significant, *F*s ≤ 2.67, *p*s ≥ 0.106, η_*p*_^2^ ≤ 0.03.

#### Feedback Effects on Quiz Performance Depending on Difficulty of Quiz Items (H5)

As in Study 1, we observed only a small overlap between the pre-study and session 1 of Study 2 regarding the solution frequency of quiz items (32.5%). Hence, we re-categorized quiz items based on their solution frequency in session 1 as easy (>67th percentile) and difficult (<33rd percentile); observed quiz scores were consequently different for easy and difficult quiz items in both feedback conditions, *t*s ≥ 2.82, *p*s < 0.006, *d*s ≥ 0.35. Then, we performed a 2 (quiz session: 2 vs. 3) × 2 (feedback condition) × 2 (difficulty of quiz items) ANOVA with quiz score as dependent variable. We found a main effect of quiz session, *F*_(1,148)_ = 84.81, *p* < 0.001, η_*p*_^2^ = 0.36, but no main effect of the difficulty of quiz items, *F*_(1,148)_ = 0.16, *p* = 0.694, η_*p*_^2^ < 0.01, and no main effect of feedback condition, *F*_(1,148)_ = 0.26, *p* = 0.614, η_*p*_^2^ < 0.01. We found a significant interaction between quiz session and difficulty of quiz items, *F*_(1,148)_ = 4.52, *p* = 0.035, η_*p*_^2^ = 0.03: Quiz scores decreased from session 2 to session 3 in case of easy quiz items, *t*(149) = −6.50, *p* < 0.001, *d* = 0.53, and in case of difficult quiz items, *t*(149) = −6.85, *p* < 0.001, *d* = 0.56. However, quiz scores for difficult items compared with quiz scores for easy items did not differ, neither in session 2, *t*(149) = −1.71, *p* = 0.089, *d* = 0.14, nor in session 3, *t*(149) = 1.61, *p* = 0.110, *d* = 0.13. All other interactions were non-significant, *F*s ≤ 1.59, *p*s ≥ 0.209, η_*p*_^2^ ≤ 0.01 (see Figure 4D). Similar to Study 1, these results provide no support for the interaction hypothesis H5.

#### Item Processing Times in a Lab and a Mobile Setting (H6)

We compared participants' item processing times observed under standardized (lab setting, *n* = 68) and unsupervised online conditions (mobile setting, *n* = 150). The analysis was limited to session 1 because feedback in terms of KRFC or AIF was only provided in this learning phase. We computed a 2 (study) × 2 (feedback condition) ANOVA. We found a main effect of study, *F*(1, 214) = 8.24, *p* = 0.005, η_*p*_^2^ = 0.04, and a main effect of feedback condition, *F*(1, 214) = 14.48, *p* < 0.001, η_*p*_^2^ = 0.06, but no interaction, *F*(1, 214) = 2.63, *p* = 0.107, η_*p*_^2^ = 0.01. Item processing times were higher in Study 1 (*M* = 16.37 s, *SD* = 5.09) than in Study 2 (*M* = 14.55 s, *SD* = 3.85), *t*(216) = 2.91, *p* = 0.004, *d* = 0.43 (H6a), and item processing times were higher for participants who received AIF (*M* = 16.20 s, *SD* = 5.19) compared with KCRF (*M* = 14.18 s, *SD* = 3.18), *t*(160.74) = 3.41, *p* = 0.001, *d* = 0.48 (H6b).

We finally checked whether the difference in item processing times between Study 1 and Study 2 during session 1 led to different quiz performances in session 2 (test phase) and in session 3 (follow-up test). The absolute performance between the two studies cannot be compared because participant samples were not identical but differently composed. Therefore, we analyzed the relative increase in short-term (quiz scores observed in session 2 minus session 1) and long-term performance (session 3 minus session 1). Notably, the short-term performance gain was similar in Study 1 (*M* = 48.46%, *SD* = 10.18) and Study 2 (*M* = 46.57%, *SD* = 9.87), *t*(216) = 1.30, *p* = 0.196, *d* = 0.19, but the long-term performance gain was significantly larger in Study 1 (*M* = 45.11%, *SD* = 10.76) than in Study 2 (*M* = 39.92%, *SD* = 9.65), *t*(216) = 3.55, *p* < 0.001, *d* = 0.52.

#### User Experience and Game Experience (H7, H8)

Replicating the result pattern of Study 1, we did not find a difference between feedback conditions regarding user experience (H7) or regarding game experience (H8) (see Table 2). The mean user experience in both feedback conditions was again above the scale's midpoint for all rating dimensions, except for dependability and novelty. In both feedback conditions, game enjoyment and game preference were above the scales' midpoints, whereas perceived competence was below the scale's midpoint.

### Discussion

Study 2 utilized a more realistic mobile setting compared to the lab setting of Study 1. Overall, we replicated the results found in Study 1. In summary, both KCRF and AIF resulted in a similar increase in quiz performance (H1) and response certainty (H2) at the short-term (session 2) and long-term (session 3) period, but quiz performance and response certainty slightly decreased from session 2 to session 3. The effect of feedback on quiz performance was not moderated by participants' initial response certainty (H3), prior knowledge (H4), and the difficulty of quiz items (H5). Similar to Study 1, quiz performance was generally higher in the group of participants who reported high (compared with low) response certainty in session 1 (learning phase), but this difference was substantially reduced in sessions 2 and 3. As in Study 1, participants with high prior knowledge showed a more temporally stable performance gain than participants with low prior knowledge. Furthermore, we observed a significant interaction between quiz session and difficulty of quiz items. However, more detailed analyses of simple main effects revealed no substantial impact of the difficulty of quiz items. The reported user experience (H7) and game experience (H8) were again relatively high and not affected by the feedback type provided.

We additionally compared Study 1 and Study 2 regarding the mean duration participants needed to process a quiz item in the learning phase (H6). Processing a quiz item included four steps: reading the question and response options, selecting an option, evaluating the response certainty, and processing the response feedback. The mean item processing time was significantly higher in Study 1 than in Study 2 by about 12% (H6a). We might speculate that this effect reflects a more focused task processing under controlled lab conditions with a lower risk of distraction and multitasking. The relative increase in quiz performance was similar in the short term (Study 1: 49% vs. Study 2: 46%), but it was larger in the long term in Study 1 (46%) compared with Study 2 (40%). Thus, the effects of feedback type on memory traces might change over time, but this interesting finding should be scrutinized by future research. Moreover, the longer processing time in case of AIF compared with KCRF (about 14%; H6b) can be explained by the more complex feedback that apparently required more processing time, given all other things being equal in the two feedback conditions.

## General Discussion

We found that the standard corrective feedback of quiz apps (KCRF) and an elaborated feedback type that provides additional information on the correct response option (AIF) had similar positive effects on the acquisition and retention of semantic knowledge. The results found under controlled lab conditions (Study 1) and a more self-regulated mobile setting (Study 2) were almost identical, indicating some generalizability across learning settings. Although Study 2 had a higher power due to the larger sample size, we overall found no difference in feedback effects.

### Effects of Mobile Quiz Apps With Different Feedback Types

We delineated why receiving AIF might be beneficial for learning as suggested by several theoretical approaches related to the encoding and retrieval of semantic knowledge (e.g., Craik and Lockhart, 1972; Anderson, 1983; Roediger and Butler, 2011). Still, our results show that AIF and the standard KCRF had similar positive effects on quiz performance and response certainty in the short and long term. These positive effects were moderated neither by participants' initial response certainty, nor by their prior knowledge, nor by task difficulty. Our results are in line with a previous study in which students scored similarly in a summative assessment after receiving delayed KCRF or elaborated feedback for problem solving (Van der Kleij et al., 2012). However, in our studies, feedback in terms of KCRF or AIF was provided immediately after responding to a question and the quiz content focused on semantic knowledge. Relatedly, providing more complex feedback did not facilitate performance in a test about text comprehension (Kulhavy et al., 1985) and providing KCRF and AIF was found to yield similar short-term retention of learning material relevant to students' regular university course (Rüth et al., 2020). Such findings might partly be due to cue overload (e.g., Karpicke et al., 2014) so that the amount of information to be encoded and stored exceeds learners' memory-related capacities. While learning took place in a self-paced manner in our studies, asking learners to take breaks after several quiz items—also known as spaced practice (see Dunlosky and Rawson, 2015a)—might be one way to reduce the risk of cue overload. Moreover, in our studies, we limited the amount of additional information (AIF) to one sentence, yet the provision of AIF for too many quiz items still might have resulted in cue overload. Importantly, cognitive (over)load induced by the complexity of the learning material can be reduced and, as a consequence, performance can increase when the learning material is presented in segments (Mayer, 2017). Hence, AIF might outperform KFRC when reducing the number of items presented in a block. More generally, it has been formulated that providing feedback with more information would enhance learning (Wisniewski et al., 2020), but this seems to remain context-specific as also illustrated by mixed findings related to problem solving (Attali and van der Kleij, 2017; Cáceres et al., 2019).

### Positive Effects of Mobile Quiz Apps in Lab and Mobile Settings

In the present studies, increases in terms of cognitive outcomes (quiz scores) and metacognitive outcomes (response certainty) were relatively long-lasting and they only slightly diminished from a subsequent test phase to an unannounced follow-up test 1 week later. Indeed, this effect of playing the quiz can be interpreted as large according to common rules of thumb for effect sizes (Cohen, 1988). Furthermore, quiz scores and response certainty followed a similar pattern and were positively correlated in each quiz session, indicating that participants' quiz scores reflected their actual knowledge level. Similar findings were found in case of retrieval tests (Barenberg and Dutke, 2019), and seem also to apply to the context of multiple-choice tests and mobile quiz apps. Notably, the benefits of quiz apps on knowledge retention that we found could also facilitate transfer effects, for instance, a better performance in other knowledge or skill domains (for a review, see Pan and Rickard, 2018). We also found a general effect of prior knowledge on long-term retention, namely, that participants with higher prior knowledge were able to retain more knowledge in the follow-up test 1 week later. Previous research found no additional benefit of elaborated feedback on problem solving or conceptual understanding given low prior knowledge, yet given high prior knowledge (e.g., Smits et al., 2008; Janelli and Lipnevich, 2021). However, participants' level of prior knowledge did not moderate the effects of AIF and KCRF. Also, across the two present studies, we found no hint for moderating effects of item difficulty and response certainty during learning on the effectiveness of feedback type. Importantly, the sample size of Study 2 was larger than the sample size of Study 1, but the overall result pattern was (nearly) perfectly replicated. Hence, missing effects of feedback type and of moderator variables do not seem to reflect simple issues of test power. Overall, our findings corroborate that quiz apps can be considered effective learning tools for the acquisition and retention of semantic knowledge, also in more self-regulated mobile settings.

Notably, most participants did not use the quiz app in a literally mobile setting (about 2–4%), but at home (about 80–83%), in a university (about 11%), or at work (about 4–5%). Therefore, we would like to highlight that feedback effects might be different if quiz apps are used in potentially more noisy mobile settings (e.g., in public transport). Participants' item processing times were longer in the lab setting than in the mobile setting, which could mean that participants in the mobile setting were less focused (Revilla and Ochoa, 2015). Supporting this notion, we found a similar short-term performance gain in both settings, but a larger long-term performance gain in the lab setting. Yet, the absolute mean item processing time was sufficiently long in both settings (lab: 16.37 s; mobile: 14.55 s), indicating that participants did not simply rush through quiz items.

### Limitations and Future Research

The results of our studies indicate that the effects of immediate KCRF and AIF are similar for the acquisition and retention of semantic knowledge. We found that these results were not moderated by learners' overall response certainty during learning, their prior knowledge, and the difficulty of quiz items. In general, moderating factors complicate the isolation of pure feedback effects (for reviews, see Mory, 2004; Shute, 2008; Van der Kleij et al., 2015; Wisniewski et al., 2020), highlighting the importance of research examining potential moderating factors.

We observed that different feedback types were similarly effective but that learners invested more time to process quiz items with AIF compared with KCRF in both studies. To gain more in-depth information on learners' memory-related processes, future studies could also use tests that ask learners to recall or recognize the content of AIF. A quiz app could also contain a function that asks learners to actively confirm that they processed the feedback information or that asks them to reflect on the feedback before proceeding to the next quiz item. To assess learners' item processing times more thoroughly, future studies could include eye movement measurements to examine learners' actual viewing times on different elements of the quiz items (Lindner et al., 2014). Still, even peripheral color cues might unfold different effects on learners' performance regarding knowledge encoding and retrieval (Gnambs et al., 2015). In our studies, the design of the quiz app was identical between feedback conditions to exclude such effects of visual elements. Moreover, the main purpose of both KCRF and AIF is to provide feedback (e.g., information about learners' performance relative to expected standards), yet learners may also use additional information in a feed-forward sense (e.g., information that allow learners to adapt their learning behavior) or in a feed-up sense (e.g., information that allow learners to specify and adjust their learning goals) (Hattie and Timperley, 2007). For instance, elaborated feedback that provides learners with explanations or examples (e.g., Janelli and Lipnevich, 2021) might be received differently by learners, such as in a feedback (e.g., student compare their performance with the required performance) or in a feed-forward sense (e.g., students memorize the provided explanations or examples, think about their appropriateness, and try to find own ones). Therefore, it seems worthwhile to complement objective measurements of performance with subjective measurements that consider how learners perceive and process feedback in learning environments.

In our studies, we examined learners' subjective experiences in terms of response certainty, user experience, and game experience. It has been discussed that response certainty should be interpreted with caution when assessing learning performance (Bush, 2015). In our case, however, quiz scores and response certainty were positively correlated across feedback conditions and studies. Of course, self-reports in performance contexts are sometimes biased, which reduces their validity (e.g., Carrell and Willmington, 1996). Still, future research should focus on potential mediation processes incorporating subjective parameters, for example, an effect of feedback type on performance via users' acceptance of feedback types. Such research on mediation effects could nicely complement the present research on moderation effects. Specifically, learners' goal orientation and their motivation to attend additional information could affect their use of elaborated feedback (Shute, 2008). Knowledge about such individual needs of learners could also allow to provide them with adaptive feedback (for a review, see Bimba et al., 2017). For instance, while participants in our studies received AIF across all quiz items regardless of the correctness and certainty of responses, elaborated feedback might be more effective than KCRF following incorrect responses (Attali and van der Kleij, 2017) or following low certainty responses (Butler et al., 2008). While such modifications might change the user experience on an item-per-item level, our results indicate that the feedback type did not affect the user experience (usability and aesthetics) on a summative level. In the present studies, participants reported high attractiveness and stimulation (interest and excitement) of the app regardless of feedback type. Examinations of subjective experiences also allow the investigation of learners' self-regulatory processes, including whether and how learners are willing to receive feedback and are seeking for feedback (cf. Hattie and Timperley, 2007). Overall, it appears to be a fruitful avenue for future research to investigate more parameters of subjective experiences (e.g., via think-aloud protocols or retrospective questions) and to integrate them into the feedback process.

Future research might also consider discussing further theoretical and practical aspects. For instance, while we referred to the feedback typology of Shute (2008), different categories of feedback types are used elsewhere (e.g., Wisniewski et al., 2020). Future research on and development of digital learning interventions could become more fruitful and comprehensible when taking into account such terminological differences and other issues that have been outlined as important (Rüth and Kaspar, 2017). Turning to more practically relevant aspects, previous works have discussed why and how testing can be an effective way of learning (see, e.g., Roediger and Karpicke, 2006; Roediger and Butler, 2011; Dunlosky et al., 2013). However, “students do use testing while learning, but results also suggest that they do not take full advantage of this effective study technique” (Dunlosky and Rawson, 2015b, p. 32). Hence, future studies could also provide more ecologically valid evidence on learners' self-regulated learning with quiz apps. For instance, which quiz apps do students and teachers prefer and for what reason? What are the effects of using student- or teacher-developed versus curated question sets? Are guidelines being considered, for instance, on the formulation of multiple-choice questions (see, e.g., Azevedo et al., 2019)? In addition to the question of choosing the most effective feedback type, practical issues also include the impact of other components of quiz apps. Overall, more research is needed to understand the effects of different features of quiz apps on learners' performance and on their subjective experiences.

### Practical Implications

The results of our studies indicate that KCRF and AIF similarly support the acquisition and retention of semantic knowledge. In this context, we can therefore not recommend the effort to add target-related information (AIF) to the standard feedback of quiz apps (KCRF). We also found that participants can focus on learning when using a quiz app in mobile settings, as indicated by the absolute item processing times. This is a crucial finding, suggesting that the use of quiz apps can foster (self-regulated) learning even in unsupervised learning settings. That participants did not perceive the quiz app as a particularly novel learning tool seems to be a negligible factor, since also in recent studies students reported to appreciate the (weekly) use of quizzes because they allowed them to receive regular feedback and to prepare for final examinations (e.g., Preston et al., 2020). Our results also indicate that the core functionality of quiz apps (without specific game-like features) is sufficient to elicit moderate to high game enjoyment and game preference. As shown by previous results, students rated the affective quality of quizzes higher compared to simulation games, and they rated the learning effectiveness and efficacy of quizzes higher compared to adventure games (Riemer and Schrader, 2015). Notably, participants in our studies used a quiz app in single player mode, while multiplayer modes might affect learning or motivational outcomes (see Abdul Jabbar and Felicia, 2015). Regarding the effectiveness of quiz apps for learning, students playing quiz games with more game-like features do not necessarily outperform students using less gamified quiz apps (Wang et al., 2016; Andzik et al., 2019; Sanchez et al., 2020). Moreover, some features such as music in quiz games might even distract students from learning (Andzik et al., 2019). It therefore seems worthwhile to consider that using a quiz app with core learning features may actually be more useful if the goal is to support student learning. More generally, testing was found to be more effective for learning than repeated exposure to learning material across learning conditions (e.g., frequency or timing of testing), learner characteristics (e.g., from preschool to adult age), learning materials (e.g., word pairs or general knowledge questions), and learning objectives (e.g., recognition or comprehension) according to meta-analyses (Rowland, 2014; Adesope et al., 2017) and reviews (Rawson and Dunlosky, 2012; Dunlosky et al., 2013). Against this background, our results emphasize that quiz apps effectively support learning via self-assessment in different settings and that even their core features can provide students a pleasant and game-like learning experience.

## General Conclusion

Corrective feedback of quiz apps (KCRF) and feedback that incorporated additional target-related information (AIF) were similarly beneficial to acquire and retain semantic knowledge in a quiz app. Learning by using the quiz app substantially increased learners' semantic knowledge in the short term and in the long term regardless of feedback type. These beneficial effects were found under controlled conditions in a lab and in an unsupervised mobile setting. Overall, quiz apps are valuable learning tools that can support remote and self-regulated learning.

## Data Availability Statement

The raw data supporting the conclusions of this article will be made available by the authors, without undue reservation.

## Ethics Statement

Ethical review and approval was not required for the study on human participants in accordance with the local legislation and institutional requirements. The participants voluntarily participated in this study and provided their written informed consent to participate in this study.

## Author Contributions

MR, JB, and KK designed the studies. MR and DZ collected the data. MR organized and supervised data collection and data curation, and programmed the software. MR and KK analyzed the data, drafted and revised the manuscript. JB and DZ revised the manuscript. JB and KK acquired funding for the studies. All authors contributed to the article and approved the submitted version.

## Conflict of Interest

The authors declare that the research was conducted in the absence of any commercial or financial relationships that could be construed as a potential conflict of interest.

## References

[B1] Abdul Jabbar A. I. Felicia P. (2015). Gameplay engagement and learning in game-based learning: a systematic review. Rev. Educ. Res. 85, 740–779. 10.3102/0034654315577210

[B2] Adesope O. O. Trevisan D. A. Sundararajan N. (2017). Rethinking the use of tests: a meta-analysis of practice testing. Rev. Educ. Res. 87, 659–701. 10.3102/0034654316689306

[B3] Anderson J. R. (1983). A spreading activation theory of memory. J. Verbal Learn. Verbal Behav. 22, 261–295. 10.1016/S0022-5371(83)90201-3

[B4] Andzik N. R. Gist C. M. Smith E. E. Xu M. Neef N. A. (2019). The effects of gaming on university student quiz performance. J. Effect. Teach. High. Educ. 2, 109–119. 10.36021/jethe.v2i1.11

[B5] Attali Y. van der Kleij F. (2017). Effects of feedback elaboration and feedback timing during computer-based practice in mathematics problem solving. Comput. Educ. 110, 154–169. 10.1016/j.compedu.2017.03.012

[B6] Azevedo J. Oliveira E. P. Beites P. D. (2019). E-assessment and multiple-choice questions: a literature review, in Handbook of Research on E-Assessment in Higher Education, eds Azevedo A. Azevedo J. (Hershey, PA: IGI Global), 1–27. 10.4018/978-1-5225-5936-8.ch001

[B7] Barenberg J. Dutke S. (2019). Testing and metacognition: retrieval practise effects on metacognitive monitoring in learning from text. Memory 27, 269–279. 10.1080/09658211.2018.1506481 30074864

[B8] Bimba A. T. Idris N. Al-Hunaiyyan A. Mahmud R. B. Shuib N. L. B. M. (2017). Adaptive feedback in computer-based learning environments: a review. Adaptive Behav. 25, 217–234. 10.1177/1059712317727590

[B9] Bjork R. A. Dunlosky J. Kornell N. (2013). Self-regulated learning: beliefs, techniques, and illusions. Ann. Rev. Psychol. 64, 417–444. 10.1146/annurev-psych-113011-143823 23020639

[B10] Boitshwarelo B. Reedy A. K. Billany T. (2017). Envisioning the use of online tests in assessing twenty-first century learning: a literature review. Res. Pract. Technol. Enhanced Learn. 12:16. 10.1186/s41039-017-0055-7 30595721 PMC6294208

[B11] Bush M. (2015). Reducing the need for guesswork in multiple-choice tests. Assess. Eval. Higher Educ. 40, 218–231. 10.1080/02602938.2014.902192

[B12] Butler A. C. Karpicke J. D. Roediger H. L. III (2008). Correcting a metacognitive error: feedback increases retention of low-confidence correct responses. J. Exp. Psychol. 34, 918–928. 10.1037/0278-7393.34.4.918 18605878

[B13] Cáceres M. Nussbaum M. González F. Gardulski V. (2019). Is more detailed feedback better for problem-solving? Interactive Learn. Environ. 1–22. 10.1080/10494820.2019.1619595

[B14] Carpenter S. K. Pashler H. Wixted J. T. Vul E. (2008). The effects of tests on learning and forgetting. Memory Cogn. 36, 438–448. 10.3758/MC.36.2.438 18426072

[B15] Carrell L. J. Willmington S. C. (1996). A comparison of self-report and performance data in assessing speaking and listening competence. Commun. Rep. 9, 185–191.

[B16] Cohen J. (1988). Statistical Power Analysis for the Behavioral Sciences, 2nd Edn. New York, NY: Routledge Academic.

[B17] Craik F. I. M. Lockhart R. S. (1972). Levels of processing: a framework for memory research. J. Verbal Learn. Verbal Behav. 11, 671–684. 10.1016/S0022-5371(72)80001-X

[B18] Dunlosky J. Rawson K. A. (2015a). Practice tests, spaced practice, and successive relearning: tips for classroom use and for guiding students' learning. Sch. Teach. Learn. Psychol. 1, 72–78. 10.1037/stl0000024

[B19] Dunlosky J. Rawson K. A. (2015b). Do students use testing and feedback while learning? A focus on key concept definitions and learning to criterion. Learn. Instr. 39, 32–44. 10.1016/j.learninstruc.2015.05.003

[B20] Dunlosky J. Rawson K. A. Marsh E. J. Nathan M. J. Willingham D. T. (2013). Improving students' learning with effective learning techniques: promising directions from cognitive and educational psychology. Psychol. Sci. Public Interest 14, 4–58. 10.1177/1529100612453266 26173288

[B21] Faul F. Erdfelder E. Lang A. G. Buchner A. (2007). G^*^Power 3: a flexible statistical power analysis program for the social, behavioral, and biomedical sciences. Behav. Res. Methods 39, 175–191. 10.3758/BF03193146 17695343

[B22] Furnham A. Monsen J. Ahmetoglu G. (2009). Typical intellectual engagement, big five personality traits, approaches to learning and cognitive ability predictors of academic performance. Br. J. Educ. Psychol. 79, 769–782. 10.1348/978185409X412147 19245744

[B23] Furnham A. Swami V. Arteche A. Chamorro-Premuzic T. (2008). Cognitive ability, learning approaches and personality correlates of general knowledge. Educ. Psychol. 28, 427–437. 10.1080/01443410701727376

[B24] Galy E. Cariou M. Mélan C. (2012). What is the relationship between mental workload factors and cognitive load types? Int. J. Psychophysiol. 83, 269–275. 10.1016/j.ijpsycho.2011.09.023 22008523

[B25] Gnambs T. Appel M. Kaspar K. (2015). The effect of the color red on encoding and retrieval of declarative knowledge. Learn. Indiv. Diff. 42, 90–96. 10.1016/j.lindif.2015.07.017

[B26] Grant M. M. (2019). Difficulties in defining mobile learning: analysis, design characteristics, and implications. Educ. Technol. Res. Dev. 67, 361–388. 10.1007/s11423-018-09641-4

[B27] Griffiths L. Higham P. A. (2018). Beyond hypercorrection: remembering corrective feedback for low-confidence errors. Memory 26, 201–218. 10.1080/09658211.2017.1344249 28671026

[B28] Hamborg K.-C. Hülsmann J. Kaspar K. (2014). The interplay between usability and aesthetics: more evidence for the “what is usable is beautiful” notion? Adv. Hum. Comput. Interaction 2014:946239. 10.1155/2014/946239

[B29] Hattie J. Timperley H. (2007). The power of feedback. Rev. Educ. Res. 77, 81–112. 10.3102/003465430298487

[B30] Hunsu N. J. Adesope O. Bayly D. J. (2016). A meta-analysis of the effects of audience response systems (clicker-based technologies) on cognition and affect. Comput. Educ. 94, 102–119. 10.1016/j.compedu.2015.11.013

[B31] Irwing P. Cammock T. Lynn R. (2001). Some evidence for the existence of a general factor of semantic memory and its components. Pers. Individ. Dif. 30, 857–871. 10.1016/S0191-8869(00)00078-7

[B32] Iterbeke K. De Witte K. Schelfhout W. (2020). The effects of computer-assisted adaptive instruction and elaborated feedback on learning outcomes. A randomized control trial. Comput. Hum. Behav. 120:106666. 10.1016/j.chb.2020.106666

[B33] Jaehnig W. Miller M. L. (2007). Feedback types in programmed instruction: a systematic review. Psychol. Record 57, 219–232. 10.1007/BF03395573

[B34] Janelli M. Lipnevich A. A. (2021). Effects of pre-tests and feedback on performance outcomes and persistence in massive open online courses. Comput. Educ. 161:104076. 10.1016/j.compedu.2020.104076

[B35] Karpicke J. D. Lehman M. Aue W. R. (2014). Retrieval-based learning: an episodic context account, in Psychology of Learning and Motivation, ed. B. H. Ross (San Diego, CA: Academic Press), 237–284. 10.1016/B978-0-12-800283-4.00007-1

[B36] Kaspar K. Hamborg K. C. Sackmann T. Hesselmann J. (2010). Die Effektivität formativer Evaluation bei der Entwicklung gebrauchs-tauglicher Software: Eine Fallstudie [The effectiveness of formative evaluation in the development of usable software: a case study]. Z. Arbeits Organisationspsychol. 54, 29–38. 10.1026/0932-4089/a000003

[B37] Kaspar K. Wehlitz T. von Knobelsdorff S. Wulf T. von Saldern M. A. O. (2015). A matter of font type: the effect of serifs on the evaluation of scientific abstracts. Int. J. Psychol. 50, 372–378. 10.1002/ijop.12160 25704872

[B38] Kim J. Y. L. Phillips T. L. (1991). The effectiveness of two forms of corrective feedback in diabetes education. J. Comput. Based Instr. 18, 14–18.

[B39] Kulhavy R. W. Stock W. A. (1989). Feedback in written instruction: the place of response certitude. Educ. Psychol. Rev. 1, 279–308. 10.1007/BF01320096

[B40] Kulhavy R. W. White M. T. Topp B. W. Chan A. L. Adams J. (1985). Feedback complexity and corrective efficiency. Contemp. Educ. Psychol. 10, 285–291. 10.1016/0361-476X(85)90025-6

[B41] Laugwitz B. Held T. Schrepp M. (2008). Construction and evaluation of a user experience questionnaire, in HCI and Usability for Education and Work. USAB 2008. Lecture Notes in Computer Science, ed Holzinger A. (Berlin; Heidelberg: Springer), 63–76. 10.1007/978-3-540-89350-9_6

[B42] Lindner M. A. Eitel A. Thoma G. B. Dalehefte I. M. Ihme J. M. Köller O. (2014). Tracking the decision-making process in multiple-choice assessment: evidence from eye movements. Appl. Cogn. Psychol. 28, 738–752. 10.1002/acp.3060

[B43] Mason B. J. Bruning R. H. (2001). Providing feedback in computer-based instruction: What the research tells us. CLASS Research Report No. 9. Center for Instructional Innovation, University of Nebraska-Lincoln.

[B44] Mayer R. E. (2017). Using multimedia for e-learning. J. Comput. Assist. Learn. 33, 403–423. 10.1111/jcal.12197

[B45] McLaughlin T. Yan Z. (2017). Diverse delivery methods and strong psychological benefits: a review of online formative assessment. J. Comput. Assist. Learn. 33, 562–574. 10.1111/jcal.12200

[B46] Merrill J. (1987). Levels of questioning and forms of feedback: instructional factors in courseware design. J. Comput. Based Instr. 14, 18–22.

[B47] Moreno R. (2004). Decreasing cognitive load for novice students: effects of explanatory versus corrective feedback in discovery-based multimedia. Instr. Sci. 32, 99–113. 10.1023/B:TRUC.0000021811.66966.1d

[B48] Moreno R. (2007). Optimising learning from animations by minimising cognitive load: cognitive and affective consequences of signalling and segmentation methods. Appl. Cogn. Psychol. 21, 765–781. 10.1002/acp.1348

[B49] Mory E. H. (2004). Feedback research revisited, in Handbook of Research on Educational Communications and Technology, ed Jonassen D. H. (Mahwah, NJ: Lawrence Erlbaum), 745–783.

[B50] Nicol D. (2007). E-assessment by design: using multiple-choice tests to good effect. J. Further High. Educ. 31, 53–64. 10.1080/03098770601167922

[B51] Nicol D. J. Macfarlane-Dick D. (2006). Formative assessment and self-regulated learning: a model and seven principles of good feedback practice. Stud. High. Educ. 31, 199–218. 10.1080/03075070600572090

[B52] Pan S. C. Rickard T. C. (2018). Transfer of test-enhanced learning: meta-analytic review and synthesis. Psychol. Bull. 144, 710–756. 10.1037/bul0000151 29733621

[B53] Pedrotti M. Nistor N. (2014). Einfluss studentischer Motivation auf die Bereitschaft zur Nutzung eines Online-Vorlesungsportals [Influence of student motivation on readiness to use an online lecture portal], in Lernräume gestalten - Bildungskontexte vielfältig denken [Designing learning spaces - thinking educational contexts diversely], ed Rummler K. (Münster: Waxmann), 332–342.

[B54] Preston R. Gratani M. Owens K. Roche P. Zimanyi M. Malau-Aduli B. (2020). Exploring the impact of assessment on medical students' learning. Assess. Eval. High. Educ. 45, 109–124. 10.1080/02602938.2019.1614145

[B55] Pridemore D. R. Klein J. D. (1995). Control of practice and level of feedback in computer-based instruction. Contemp. Educ. Psychol. 20, 444–450. 10.1006/ceps.1995.1030

[B56] Rawson K. A. Dunlosky J. (2012). When is practice testing most effective for improving the durability and efficiency of student learning? Educ. Psychol. Rev. 24, 419–435. 10.1007/s10648-012-9203-1

[B57] Revilla M. Ochoa C. (2015). What are the links in a web survey among response time, quality, and auto-evaluation of the efforts done? Soc. Sci. Comput. Rev. 33, 97–114. 10.1177/0894439314531214

[B58] Riemer V. Schrader C. (2015). Learning with quizzes, simulations, and adventures: students' attitudes, perceptions and intentions to learn with different types of serious games. Comput. Educ. 88, 160–168. 10.1016/j.compedu.2015.05.003

[B59] Roediger H. L. III Agarwal P. K. McDaniel M. A. McDermott K. B. (2011). Test-enhanced learning in the classroom: long-term improvements from quizzing. J. Exp. Psychol. 17, 382–395. 10.1037/a0026252 22082095

[B60] Roediger H. L. III Butler A. C. (2011). The critical role of retrieval practice in long-term retention. Trends Cogn. Sci. 15, 20–27. 10.1016/j.tics.2010.09.003 20951630

[B61] Roediger H. L. III Karpicke J. D. (2006). Test-enhanced learning: taking memory tests improves long-term retention. Psychol. Sci. 17, 249–255. 10.1111/j.1467-9280.2006.01693.x 16507066

[B62] Rowland C. A. (2014). The effect of testing versus restudy on retention: a meta-analytic review of the testing effect. Psychol. Bull. 140, 1432–1463. 10.1037/a0037559 25150680

[B63] Rüth M. Breuer J. Morten T. Kaspar K. (2020). Bedeutet mehr Feedback auch mehr lernen? Die Wirkung von erweitertem und korrigierendem Feedback in einem digitalen Quizspiel auf die Lernleistung [More feedback, more learning? The effect of elaborated and corrective feedback in a digital quiz game on learning performance], in Bildung, Schule und Digitalisierung [*Education, School, and Digitalization*], eds Kaspar K. Becker-Mrotzek M. Hofhues S. König J. Schmeinck D. (Münster: Waxmann), 25–30.

[B64] Rüth M. Kaspar K. (2017). The e-learning setting circle: first steps toward e-learning theory development. Electron. J. e-Learn. 15, 94–103.

[B65] Ryan R. M. Rigby C. S. Przybylski A. (2006). The motivational pull of video games: a self-determination theory approach. Motiv. Emotion 30, 344–360. 10.1007/s11031-006-9051-8

[B66] Sanchez D. R. Langer M. Kaur R. (2020). Gamification in the classroom: examining the impact of gamified quizzes on student learning. Comput. Educ. 144:103666. 10.1016/j.compedu.2019.103666

[B67] Schipolowski S. Wilhelm O. Schroeders U. (2014). On the nature of crystallized intelligence: the relationship between verbal ability and factual knowledge. Intelligence 46, 156–168. 10.1016/j.intell.2014.05.014

[B68] Seebauer S. (2014). Validation of a social media quiz game as a measurement instrument for climate change knowledge. Entertain. Comput. 5, 425–437. 10.1016/j.entcom.2014.10.007

[B69] Shute V. J. (2008). Focus on formative feedback. Rev. Educ. Res. 78, 153–189. 10.3102/0034654307313795

[B70] Sitzman D. M. Rhodes M. G. Tauber S. K. (2014). Prior knowledge is more predictive of error correction than subjective confidence. Memory Cogn. 42, 84–96. 10.3758/s13421-013-0344-3 23797971

[B71] Smits M. H. Boon J. Sluijsmans D. M. Van Gog T. (2008). Content and timing of feedback in a web-based learning environment: effects on learning as a function of prior knowledge. Interactive Learn. Environ. 16, 183–193. 10.1080/10494820701365952

[B72] Spanjers I. A. Könings K. D. Leppink J. Verstegen D. M. de Jong N. Czabanowska K.. (2015). The promised land of blended learning: quizzes as a moderator. Educ. Res. Rev. 15, 59–74. 10.1016/j.edurev.2015.05.001

[B73] Sperring S. Strandvall T. (2008). Viewers' experiences of a TV quiz show with integrated interactivity. Int. J. Hum. Comput. Interact. 24, 214–235. 10.1080/10447310701821590

[B74] Swart E. K. Nielen T. M. J. Sikkema-de Jong M. T. (2019). Supporting learning from text: a meta-analysis on the timing and content of effective feedback. Educ. Res. Rev. 28:100296. 10.1016/j.edurev.2019.100296

[B75] Tavakol M. Dennick R. (2011). Making sense of Cronbach's alpha. Int. J. Med. Educ. 2, 53–55. 10.5116/ijme.4dfb.8dfd 28029643 PMC4205511

[B76] Tsai F. H. Tsai C. C. Lin K. Y. (2015). The evaluation of different gaming modes and feedback types on game-based formative assessment in an online learning environment. Comput. Educ. 81, 259–269. 10.1016/j.compedu.2014.10.013

[B77] Van der Kleij F. M. Eggen T. J. H. M. Timmers C. F. Veldkamp B. P. (2012). Effects of feedback in a computer-based assessment for learning. Comput. Educ. 58, 263–272. 10.1016/j.compedu.2011.07.020

[B78] Van der Kleij F. M. Feskens R. C. Eggen T. J. H. M. (2015). Effects of feedback in a computer-based learning environment on students' learning outcomes: a meta-analysis. Rev. Educ. Res. 85, 475–511. 10.3102/0034654314564881

[B79] Van der Kleij F. M. Timmers C. F. Eggen T. J. H. M. (2011). The effectiveness of methods for providing written feedback through a computer-based assessment for learning: a systematic review. CADMO 19, 21–38.

[B80] Wang A. I. Zhu M. Sætre R. (2016). The effect of digitizing and gamifying quizzing in classrooms, in Proceedings of the 10th European Conference on Games Based Learning, eds Connolly T. Boyle L. (Sonning Common: Academic Conferences and Publishing International), 729–737.

[B81] Wisniewski B. Zierer K. Hattie J. (2020). The power of feedback revisited: a meta-analysis of educational feedback research. Front. Psychol. 10:3087. 10.3389/fpsyg.2019.03087 32038429 PMC6987456

[B82] Zwarun L. Hall A. (2014). What's going on? Age, distraction, and multitasking during online survey taking. Comput. Hum. Behav. 41, 236–244. 10.1016/j.chb.2014.09.041

